# Patterns of Antibiotic Resistance in *Enterobacteriaceae* Isolates from Broiler Chicken in the West Region of Cameroon: A Cross-Sectional Study

**DOI:** 10.1155/2022/4180336

**Published:** 2022-06-09

**Authors:** Jude Fonbah Leinyuy, Innocent Mbulli Ali, Ousenu Karimo, Christopher B. Tume

**Affiliations:** ^1^Research Unit of Microbiology and Antimicrobial Substances, Department of Biochemistry, University of Dschang, P.O. Box 67, Dschang, Cameroon; ^2^The Biotechnology Centre, University of Yaoundé I, P.O. Box 3851, Yaoundé, Cameroon; ^3^Department of Biochemistry, Faculty of Science, University of Bamenda, P.O. Box 39, Bambili, Cameroon

## Abstract

**Background:**

The emergence of multidrug-resistant food-borne pathogens of animal origin including *Enterobacteriaceae* is a growing concern. Identifying and monitoring resistance in isolates from human-related environments are of clinical and epidemiological significance in containing antimicrobial resistance. This study aimed to contribute towards the fight against antibiotic resistance and ameliorate the management/treatment of *Enterobacteriaceae*-linked diseases in Cameroon.

**Methods:**

Cloacal swabs from healthy broilers were enriched in buffered-peptone-water and cultured on EMB agar. Antibiotic susceptibility was tested on Mueller-Hinton-Agar by disc diffusion. Plasmid-borne genes for extended-spectrum beta lactamase (ESBL) and resistance to Quinolones (PMQR) and Aminoglycosides were detected by standard endpoint polymerase chain reaction (PCR).

**Results:**

A total of 394 isolates were identified belonging to 12 *Enterobacteriaceae* genera, the most prevalent were *Escherichia coli* (81/394 = 20.56%), *Salmonella* spp (74/394 = 18.78%), and *Klebsiella* spp (39/394 = 9.90%) respectively. Overall, 84/394 (21.32%) were ESBL producers, 164/394 (41.62%) were resistant to quinolones, 66/394 (16.75%) resistant to aminoglycosides with 44.0% (173/394) expressing MDR phenotype. Poor hygiene practice (OR 2.55, 95% CI: 1.67, 3.89, *p*=0.001) and rearing for >45 days, (OR = 7.98, 95% CI: 5.05, 12.6, *p*=0.001) were associated with increased carriage of MDR. Plasmid-borne resistance genes were detected in 76/84 (90.48%) of ESBL-producing isolates, 151/164 (92.07%) quinolone resistant isolates and 59/66 (89.39%) aminoglycoside resistant isolates with co-occurrence of two or more genes per isolate in 58/84 (69.05%) of ESBLs, 132/164 (80.49%) of quinolone resistant isolates and 28/66 (42.42%) of aminoglycoside resistant isolates.

**Conclusion:**

This study found high carriage and widespread distribution of *Enterobacteriaceae* with ESBL and MDR in broiler chicken in the West Region of Cameroon. Most PMQR genes in bacteria were found at levels higher than is seen elsewhere, representing a risk in the wider human community.

## 1. Introduction

*Enterobacteriaceae* is a heterogeneous Family of Gram negative non-spore forming bacilli which primarily live in guts of humans and other higher animals where they can be pathogens or members of the normal intestinal microflora but are also widely distributed in soil, water, and decaying matter [1, 2]. Many members of this Family are clinically important pathogens with extensive capacity to develop antibiotic resistance (ABR) due to a myriad of plasmid-borne resistance genes and a high capacity of mutation in the phase of environmental stress such as in the presence of antibiotics [3–5]. These have made *Enterobacteriaceae* a serious threat to public health due to the association of different resistance mechanisms and the insufficient development of new drugs which make these microorganisms resistant to almost all available antibiotics [6].

The World Bank multiple indicators cluster survey (MICS5), 2014 shows that in Cameroon, diarrheal diseases (*Enterobacteriaceae* being a main cause) account for 5,01% of annual deaths; being the 5th cause of mortality after malaria, HIV-AIDS, neonatal diseases, and lower respiratory diseases [7]. The country's present antimicrobial resistance (AMR) surveillance targets only priority diseases including malaria, HIV-AIDS, and *tuberculosis* but the development of multidrug resistance (MDR) in *Enterobacteriaceae* as reported in local studies on human and animal health and the environment is acknowledged by the Ministry of Health [8] with among others, an MDR of 39.8% in *Salmonella* serotypes from chicken in retail markets in Yaoundé gotten by Wouafo et al. [9] and an increase in the resistance by *E. coli* to reference antibiotics against *Enterobacteriaceae* including cephalosporins, fluoroquinolones and aminoglycosides within the period from 2009 to 2013 noted by Longla et al. at the Yaoundé University Teaching Hospital [10]. However, the National Antimicrobial Resistance Action Plan 2018–2020 envisaged the surveillance of pathogenic *Enterobacteriaceae* based on WHO criteria of timely detection, reporting, risk assessment and monitoring of emerging ABR including *Shigella* spp, Salmonella spp, *E. coli* and *K. pneumoniae* [8]. It is thus of epidemiological importance to monitor the trends of resistance in these bacteria isolated from human-related environment such as food animals so as to contain the vicious cycle whereby human activities induce the development of ABR in bacteria which in turn have repercussions on human health [11].

The area of study, the West Region of Cameroon is intensively involved in animal husbandry, mainly poultry and pig farming which provide food and jobs to a great number of people; these activities highly linked to food-borne infections [12, 13]. We focus on chicken, an important source of contamination with clinically important human pathogens [14] and a main source of meat for households and mass catering events in the Region known for its attachment to cultural celebrations including burial and funeral celebrations [15]. They tend to harbour extensive numbers and diverse types of enteric bacteria both commensal and pathogenic [16]. Such co-occurrence coupled with abuse of antibiotics in Cameroonian poultries [17] can give opportunity for sharing of resistance genes among species by means of mobile genetic elements (MGEs) and chromosomal recombination due to stress from antibiotics leading to emergence of resistant and multidrug resistant strains [3, 4, 16].

When AMR develops in commensal intestinal microflora it tends to go unchecked making these commensals to act as reservoir of AMR [16]. Thus the development of AMR in commensal *Enterobacteriaceae* of animal origin is a measure for early detection of AMR in the community [18]. There is therefore need for thorough research on AMR as a contribution to the country's development plans. The few studies carried out in the West Region have been centred on human isolates and clinically important *Enterobacteriaceae* and also at point locations [19–21]. With the lack of epidemiological data on prevalence and ABR among *Enterobacteriaceae*, pathogens and commensals alike in human-related environment, chicken being one of the main meat types in Cameroon [22] and an important source of *Enterobacteriaceae* infection for humans [14], and bearing in mind that the development of ABR in commensal *Enterobacteriaceae* of animal origin is a measure for early detection of ABR in the community [18], our quest for patterns of ABR in *Enterobacteriaceae* in the West Region of Cameroon prompted us to use chicken as our sample source. This research aimed to uncover the extent of the problem of antimicrobial resistance and its risk factors among poultry farming community in West region, noted as the production bay in Cameroon. It is hoped that information gathered would call the attention of stakeholders, including veterinarians, physicians, microbiologists, livestock producers, public health workers and relevant government agencies to the need for basic salvaging measures to curb the development and dissemination of ABR and contribute towards the management of *Enterobacteriaceae*-linked diseases.

## 2. Materials and Methods

### 2.1. Aim

The aim of this study was to contribute towards the fight against the development of ABR so as to ameliorate the management and treatment of diseases linked to *Enterobacteriaceae* in Cameroon; providing epidemiological data on prevailing *Enterobacteriaceae* and their resistance patterns and risk factors.

### 2.2. Study Design

The study was a cross-sectional study which ran from October 2018 to September 2021, punctuated from January 2020 to January 2021 due to the COVID-19 pandemic. October 2018 to December 2020 was field work and sampling, laboratory isolation and characterisation, and antibiotic susceptibility testing. February 2021 to September 2021 was molecular analysis of antibiotic resistance genes.

### 2.3. Study Site

**Figure 1:**
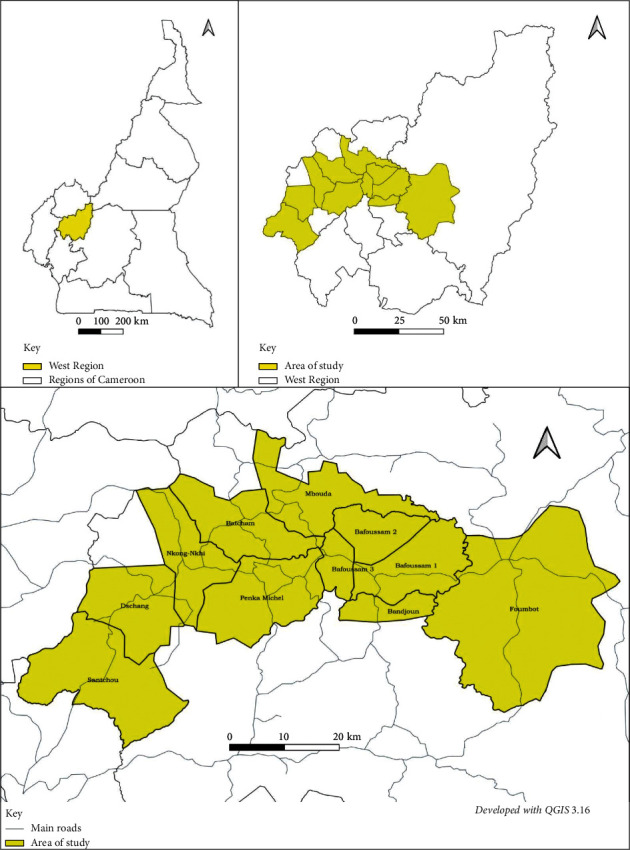
Localisation of the study area.

The West Region with an area of 13,892 km^2^ is a territory located in the central-western portion of the Republic of Cameroon located at 5°30′N 10°30′E. It shares borders with the North West Region to the northwest, the Adamaoua Region to the northeast, the Centre Region to the southeast, the Littoral Region to the southwest, and the Southwest Region to the west. The West Region is the smallest of Cameroon's ten regions in area, yet it has the second highest population density; a population of 1,865,394 (2013) with density of 142.9 inhabitants/km^2^ as of 2017 [23, 24]. Its Capital is Bafoussam in the Mifi Division. It has 8 Divisions: Bamboutos, Upper-Nkam, Upper-Plateau, Koung-Khi, Ménoua, Mifi, Ndé and Noun [24]. The Region is found in the Grass field plateaus of the Western Highlands with a cold climate and the main ethnic groups are the Bameliké and Bamoum [25].  Figure 1 below shows the localisation of the study site.

Laboratory work was carried out at the Research Unit of Microbiology and Antimicrobial Substances (RUMAS) at the University of Dschang.

### 2.4. Sample Collection

The study was performed on cloacal swabs from healthy broiler chicken irrespective of age from 28 farms in 11 locations in 5 out of the 8 Divisions of the West Region of Cameroon. We used stratified sampling with Divisions as the strata.

Cloacal swabs were collected by inserting a sterile swab into the cloaca. Each swab was immersed in 5 ml of a sterile physiological solution in swab sampling tubes. The samples were transported in cool thermo-flasks to the laboratory for analysis [26].

### 2.5. Sample Enrichment and Culture

The swabs were dissolved in 10 ml of buffered peptone water and incubated for 24 hrs at 37°C. The enriched samples were cultured on EMB agar by plate streaking and incubated for 48 hours [1, 27].

### 2.6. Isolation of Isolates and Preservation

Isolated colonies on the EMB agar were identified based on colony characteristics, picked and conserved in a conservation medium; a mixture of glycerol and Muller Hilton broth at 1 part to 3 parts and stored in a refrigerator at −20°C [28, 29].

### 2.7. Phenotypic Characterisation of Isolates

Isolates were identified using colony characteristics on the culture medium (EMB agar) and 10 rapid screening biochemical tests [1,30] locally used in routine clinical laboratory diagnosis including: glucose and lactose fermentation, hydrogen sulphide (H_2_S) and gas production using Kligler Iron Agar (KIA), urease activity and indole production using urea broth and Kovacs' reagent, mannitol fermentation and motility using mannitol agar, citrate fermentation using citrate agar and catalase test.

### 2.8. Antibiotic Susceptibility Testing

Fresh colonies were used to prepare 0.5 McFarland solution inoculums using sterile physiological water. Antibiotic susceptibility testing was carried out on Mueller Hilton Agar by the Kirby-Bauer disc diffusion method [2, 30]. Screening for ABR in the isolates was done using the following antibiotics in Table 1 [31], and resistant control strains (ATCC stains of *E. coli*, *K. pneumoniae* and *Salmonella* Typhi) maintained by the pharmacology research group of the RUMAS.

Phenotypic determination of resistance by penicillinase production was tested by combination disc test with amoxicillin/clavulanic acid with comparison to amoxicillin. Extended spectrum beta-lactamase production was tested by observing resistance to amoxicillin followed by resistance to ceftriaxone or cefotaxime [32]. Confirmation of ESBL production was done by double disc synergy test on MHA by placing a disk of amoxicillin-clavulanic acid at the centre surrounded by discs of cefotaxime, ceftriaxone and ceftazidime 2 cm apart and observing for a clear zone of intersection between the central and peripheral discs [33, 34].

### 2.9. Detection of Resistance Genes

DNA extraction from fresh colonies was done by heat shock. A loop-full of a fresh colony was dissolved in 400 *μ*l of Tris-EDTA 1X buffer (Tris-Cl 0.1 M and EDTA 0.01 M diluted 1/10). The solution was vortexed for 5 s and heated in a water bath at 95°C for 25 min. The heated solution was centrifuged at 13000 tpm for 3 min and the supernatant containing DNA was extracted and used for PCR [35].

Amplification of some representative epidemiologically important plasmid-borne resistance genes including ESBL genes, PMQR genes and plasmid-mediated aminoglycoside resistance (PMAR) genes was done using the following oligonucleotides and reaction conditions in Table 2:

PCR was done with a 25 *μ*l reaction mix composed of 14.9 *μ*L of PCR grade water, 2.5 *μ*L of 1X standard Taq buffer solution with 2.5 mM MgCl2, 1 *μ*L of forward primer, 1 uL of reverse primer, 0.5 *μ*L of DNTP mix, 0.1 *μ*L of standard Taq and 5 *μ*L of DNA solution in a TECHNE® thermocycler [40]. Reaction products were migrated on a 1.5% agarose gel and revealed under UV light.

### 2.10. Data Analysis

This research work generated information on *Enterobacteriaceae* carriage in broilers, identification of *Enterobacteriaceae* organisms, their prevalence, their antibiotic susceptibility profiles (susceptible, resistant, multidrug resistant and ESBL producing) and, odds ratios and correlations between outcomes (*Enterobacteriaceae* carriage, prevalence, resistance, MDR and ESBL production) and risks.

*Enterobacteriaceae* carriage was determined by the count of the different colonies on the *Enterobacteriaceae*-specific growth medium (EMB agar) based on colony aspect and morphology. The identity of the organism was gotten by interpreting the colony aspect and the results of the phenotypic reactions using the table of reactions below in Table 3 [41–44].

Susceptibility to antibiotics was evaluated using standard values as given by Sigma Aldrich® [45]. MDR was assessed as resistance of an isolate to two or more antibiotics belonging antibiotic classes of choice used against *Enterobacteriaceae* (cephalosporins, carbapenems, quinolones or aminoglycosides). ESBL production was interpreted from double disc synergy test between amoxicillin/clavulanic acid disc and ceftriaxone, ceftazidime or cefotaxime disc.

Arithmetic operations and conversions were done using Microsoft Excel sheets while frequencies, prevalence, odds ratios, correlations and diagrams were done using IBM SPSS Statistics 20.

## 3. Results

### 3.1. Evaluation of Risks in Farms

Samples were collected from 28 farms in 11 locations in 5 out of the 8 Divisions in the West Region. Evaluating risks, 25 farms out of 28 (89.29%) used antibiotics on their chicken, 138/275 (50.18%) subjects sampled were more than 30 days old and fell on the category “old” while 137/275 (49.18%) were 30 days old or less and fell in the category “young”, 7 farms out of 28 (25.00%) regularly cleaned feeders and drinkers for the chicken, 12 farms out of 28 (42.86%) gave unsure water to their chicken and 18 out of 28 farms (64.29%) had dirty environment with or without stagnant sewage.  Table 4 below gives information on the locations of farms, number of samples and isolates and the risk factors to which chicken subjects were exposed.

**Figure 2:**
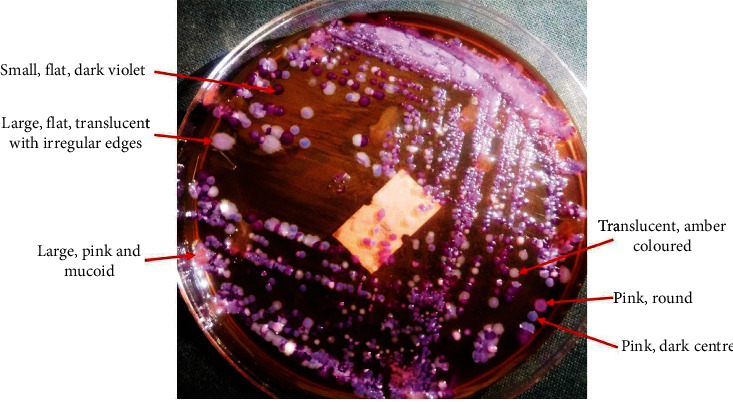
A culture plate showing bacterial carriage in a sample. At least six different colony types can be identified.

A total of 275 cloacal swabs were collected. All these samples tested positive for *Enterobacteriaceae* with a carriage of at least 2 different colony types and a mean of 3 different colony types per sample.  Figure 2 below shows a bacterial carriage of 6 different colony types in one sample.

Bacterial carriage of greater than or equal to 3 different colony types present in culture correlated significantly with the 3 environmental risk factors evaluated as shown in Table 5 below.

### 3.2. Prevalence of Members of the Enterobacteriaceae

**Figure 3:**
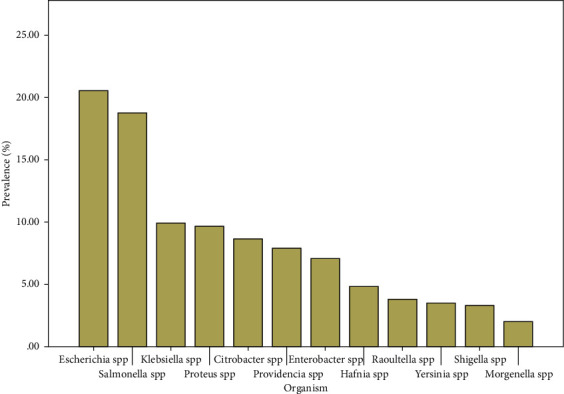
Prevalence of isolates of different *Enterobacteriaceae* species.

From the 275 cloacal swabs collected, a total, 394 isolates were obtained and characterised belonging to 12 different genera of *Enterobacteriaceae*. The 394 isolates were distributed as 81 (20.56%) *Escherichia* spp, 74 (18.78%) *Salmonella* spp, 39 (9.90%) *Klebsiella* spp, 38 (9.64%) *Proteus* spp, 34 (8.63%) *Citrobacter* spp, 31 (7.87%) *Enterobacter* spp, 28 (7.11%) *Providencia* spp, 19 (4.82%) *Hafnia* spp, 15 (3.81%) *Shigella* spp, 14 (3.55%) *Raoultella* spp, 13 (3.30%) *Yersinia* spp and 8 (2.03%) *Morgenella* spp. We note the outstanding predominance of *Escherichia* spp and *Salmonella* spp as shown in Figure 3 below.

Detailed information on the prevalence at each sampling site is given in Table 6 below as percentages only to render the information less cumbersome. The computed coefficient of variation of the prevalence of each organism in the various locations showed relatively uniform prevalence across the study area.

### 3.3. Prevalence of Antibiotic Resistance

Antibiotic susceptibility testing on the 394 isolates showed the following overall resistance to the various antibiotics tested: amoxicillin 345 (87.8%), amoxicillin/clavulanic acid 227 (57.8%), ceftriaxone 79 (20.1%), cefotaxime 65 (16.5%), imipenem 16 (4.1%), gentamicin 58 (14.5%), amikacin 12 (3.1%), ciprofloxacin 142 (37.1%), levofloxacin 124 (33.1%), doxycycline 380 (96.7%).

There were 230 (58.38%) of isolates resistant to at least one antibiotic class of choice against *Enterobacteriaceae* (cephalosporins, carbapenems, quinolones or aminoglycosides). 80 (20.3%) of the isolates were resistant to at least one of the cephalosporins tested, 164 (41.62%) were resistant to at least one of the quinolones tested and 66 (16.75%) were resistant to at least one of the aminoglycosides tested. 232 (58.88%) of the isolates showed penicillinase production but ESBL production was much lower with 84 (21.32%) and 173 (44.0%) showed MDR when considering only beta lactams, quinolones and aminoglycosides, but adding the contribution of tetracyclines MDR rose to 85.53% (337 isolates).  Table 7 below gives details of the prevalence of ABR in various isolates given as percentages only to make the information less cumbersome.

**Figure 4:**
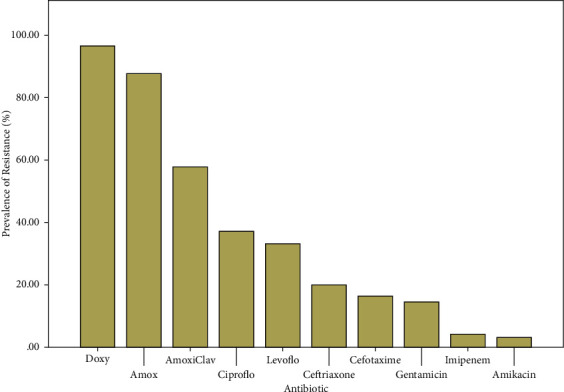
General prevalence of resistance of isolates to various antibiotics tested.

**Figure 5:**
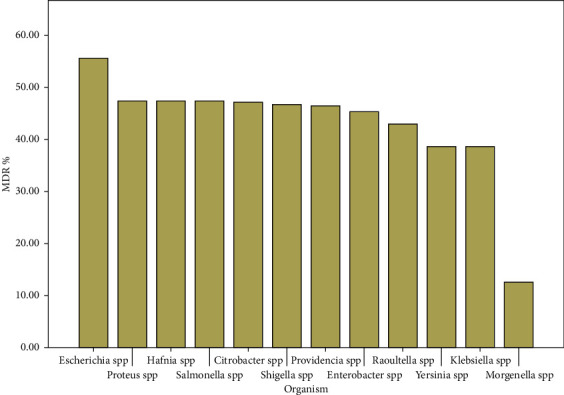
General prevalence of MDR amongst the isolates.

The overall prevalence of resistance and MDR are presented in Figures 4and 5 below.

**Figure 6:**
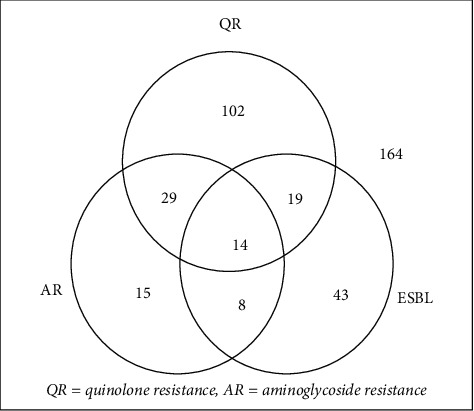
Association of various resistance phenotypes among isolates.

The association of resistance phenotypes to the various antibiotic classes is shown in Figure 6 below.

Figure 6 shows that 230 out of 394 isolates (58.38%) were at least either ESBL producer, quinolone resistant or aminoglycoside resistant, an association of two or of all the 3 resistances studied. It also shows that the number of ESBL producing isolates that were quinolone resistant was significantly lower than the number of isolates that were not quinolone resistant (32/84 against 52/84 respectively) while the number of aminoglycoside isolates that were resistant to quinolones was significantly higher than the number of isolates that were not quinolone resistant (43/66 against 23/66).

### 3.4. Association of Risk Factors among Chickens with Antibiotic Resistance

The outcome “resistance” indicates isolates showing resistance to at least one antibiotic from one class of choice. The development of resistance to at least one antibiotic class correlated significantly to the age of the chicken (*p*=0.001, OR = 13.491) and food hygiene (*p*=0.007, OR = 1.783). Isolates had higher risk of developing resistance on exposure to all risks except unsure water. ESBL production correlated significantly to age of chicken (*p*=0.001, OR = 4.505) and isolates had higher risk of developing ESBL on exposure to all risks except use of antibiotics. MDR correlated significantly to the age of the chicken (*p*=0.001, OR = 7.980) and isolates had a higher risk of developing MDR on exposure to all risks except unsure water. The risk “use of antibiotics” was quasi constant thus did not correlate with outcomes but increased the chances of developing the various outcomes as seen in the OR greater than 1. The association of risks to resistance outcomes is presented in Table 8 below.

### 3.5. Detection of Plasmid-borne Resistance Genes in Isolates

The prevalence of some representative clinical and epidemiologically important plasmid-borne genes in the phenotypically resistant isolates as detected by PCR are presented per resistance to each antibiotic class in the subsections below. Added are the details about 3 most prevalent and local clinically important genera; *E. coli*, *Salmonella* and *Klebsiella*. Worth noting here also is the relatively high occurrence of the resistance genes in clinically less important isolates under the group “Others”.

No plasmid-borne resistance genes were detected in 8 out of the 84 (9.5%) ESBL producing isolates, 13 out of the 164 (7.9%) quinolones resistant isolates and 7 out of the 66 (10.6%) aminoglycosides resistant isolates.

#### 3.5.1. Detection of Plasmid-borne Beta Lactamase Genes

Six beta lactamase genes were amplified as shown in Table 9 below. The *blaTEM-1* gene was the most prevalent gene among beta lactamase producers in 59/84 (70.24%) of the isolates.

**Figure 7:**
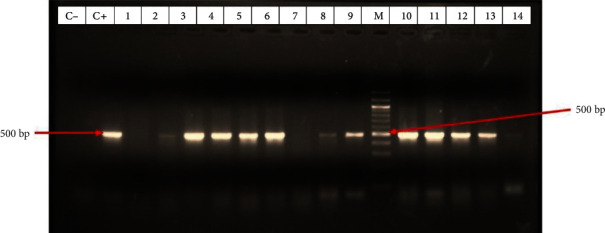
Gel image showing the amplification of the *blaTEM-1* gene fragment at 500 bp.

Figure 7 below is a sample gel image showing the amplification of gene fragment of the *blaTEM-1* gene, one of the beta lactamase genes amplified by PCR.

#### 3.5.2. Detection of PMQR Genes

Five PMQR genes were amplified as shown in Table 10 below. The *aac (6′)-IB-CR*, a variant of the aminoglycoside resistance gene that confers resistance to quinolones was the most prevalent gene among PMQR genes in 97/164 (59.15%) of the isolates.

**Figure 8:**
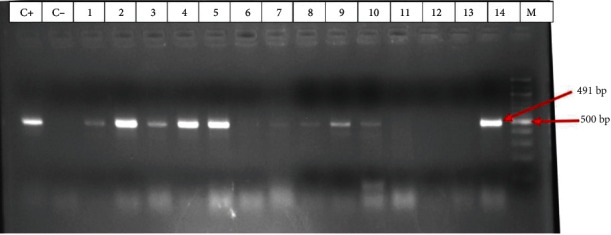
Gel image showing the amplification of the *qnrS* gene fragment at 491 bp.

Figure 8 below is a sample gel image showing the amplification of a fragment of the *qnrS* gene, one of the PMQR genes amplified by PCR.

#### 3.5.3. Detection of PMAR Genes

Three PMAR genes were amplified as shown in Table 11 below. The *aac(6′)-IB* gene was the most prevalent gene among PMAR genes in 51/66 (77.27%) of the isolates.

**Figure 9:**
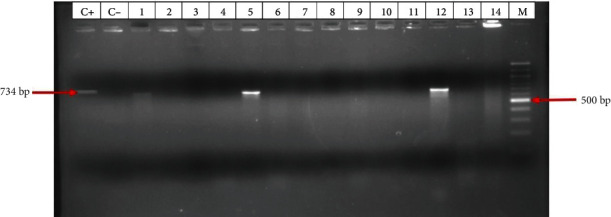
Gel image showing the amplification of the aph(3′)-IA gene fragment at 734 bp.

Figure 9 below is a sample gel image showing the amplification of a fragment of the *aph(3′)-IA* gene, one of the PMAR genes amplified by PCR.

### 3.6. Co-occurrence of Plasmid-borne Resistance Genes Enterobacteriaceae

The Co-occurrence of the Various Plasmid-borne Genes as Detected by PCR Is Summarized in Table 12 below.

## 4. Discussion

In this study, we sought to determine the extent and distribution of antimicrobial resistance phenotypes and detect plasmid-mediated genes associated with ESBL production, Quinolone and Aminoglycoside resistance among broilers in poultry farms in the West region of Cameroon. We also identified risk factors that were associated with the carriage of multidrug resistant forms of the isolates across the study area.

In total, 394 isolates were detected in all samples analysed. There was a high prevalence of the genera *Escherichia* 81/394 (20.56%) and *Salmonella* 74/394 (18.78%) among the isolates followed by *Klebsiella* 39/394 (9.90%). These genera have clinically important species causing various diseases in human [46] thus the need to monitor ABR in these bacteria. The isolation of members such as *Shigella* even in few numbers is already a cause for concern given their potential to cause epidemics when virulent. However, the prevalence is similar to what is found elsewhere in several studies [47, 48]. The fairly uniform prevalence of the organisms in locations studied can be explained by the uniform socio-demographic and geographical nature (grass field highlands) of the area [25].

Overall, the *Enterobacteriaceae* organisms isolated showed a high level of resistance to amoxicillin 345/394 (87.8%) of the group of Penicillins and doxycycline 380/394 (96.7%) of the class of Tetracyclines. These results were concordant with results obtained in 2013 by Tago et al. in Ivory Coast, a similar geographic area to Cameroon [48] and further confirmed that these are no more drug classes of choice against *Enterobacteriaceae*. Traditionally *Enterobacteriaceae* have developed high resistance to Penicillins via production of penicillinase [49] this explains high resistance to amoxicillin. As seen in similar studies, *E. coli* and *Klebsiella* maintained their known high production of ESBLs [50] with 24.69% and 30.77% respectively compared to the general 21.32%. Generally, among the drug classes of choice prescribed against *Enterobacteriaceae*, isolates showed a higher resistance to Quinolones [142/394 (37.1%) for ciprofloxacin and 124/394 (33.1%) for levofloxacin] compared to third generation Cephalosporins [79/394 (20.1%) for ceftriaxone and 65/394 (16.5%) for cefotaxime], and Aminoglycosides [12/394 (3.1%) for amikacin and 58/394 (14.5%) for gentamicin]. Compared to early reports such as that of Robert et al. 2001 [51], resistance to quinolones is on the rise with for example ciprofloxacin 10% (90% susceptibility) in 1998 to 142/394 (37.1%) in the present study. The high resistance to ciprofloxacin should be monitored closely because due to its availability in tablet form, it is one of the highest prescribed and self-medications taken in simple *Enterobacteriaceae* related diseases and gastroenteritis in the West Region and most of parts of Cameroon owing to rampant drug misuse and self-medication which is not only in clinical settings but also in poultry farms [17, 19, 20, 52].

Multidrug resistance was observed to be high in all the genera with an overall prevalence of 337/394 (85.53%). This is similar to the trend seen elsewhere as reported by Leski et al. [53]. However, considering that Tetracyclines are not drugs of choice against *Enterobacteriaceae* [54], ignoring the contribution of doxycycline this value falls considerably to 173/394 (44.0%). This high MDR prevalence in mostly commensal organisms like *Escherichia* and *Proteus* [219/394 (55.56%) and 187/394 (47.37%) respectively] may go unchecked but in the face of an opportunistic infection, the treatment becomes difficult due to the developed MDR [55]. ESBL production was observed at fairly moderate levels but the co-development of MDR and ESBL further compounds the failure of antibiotics in disease treatment [56]. A bacterial carriage of an average of 3 different *Enterobacteriaceae* colony types was found in each sample indicating the possibility of the development of ABR and MDR through horizontal gene transfer by means of MGEs such as plasmids [17, 30].

This research showed from the analysis of the odds ratios of risks that poor sanitation at poultry farm, from environment to feeding predisposed chicken first to high bacterial carriage which predisposed these bacteria to developing resistance and MDR. The sanitary conditions in the poultries visited were average, not up to standard conditions mainly due to the nature of construction and materials used which promoted poor hygiene around the poultry farms. Some areas did not have pipe borne water thus farmers used well water with doubtful cleanliness. These are conditions that usually favour extensive infection of the animals [57, 58] and this was confirmed by observations made in the current study which found an increase in the odds of infection of chicken among farms with poor feeding hygiene practice and poor sanitary practice in poultry farm.

Though not significant, antibiotic use was seen to be a risk factor with OR 1.40. This is because almost all poultry farms 26/28 (92.86%) used antibiotics regularly making it a statistical constant, as reported by Guetiya et al. [52]. Correlation of these outcomes with long rearing periods (>45 days) could be explained by the long duration of exposure to the risks [59], the time to get infected or for co-infection to allow horizontal gene transfers.

This research also showed that ESBL producing isolates that were quinolone resistant were significantly lower than those that were not quinolone resistant (32/84 against 52/84 respectively) while aminoglycoside isolates that were resistant to quinolones were significantly higher than those that were not quinolone resistant (43/66 against 23/66). This suggests that quinolone resistance could not be largely plasmid-mediated because plasmid-carried quinolone resistance genes tend to occur with ESBL producing genes creating the opposite scenario [56]. This observation thus suggests other mechanisms of resistance such as a chromosomal DNA based quinolone resistance could be participating due to antibiotic misuse. López et al. showed that ciprofloxacin induced chromosomal recombinations in *E. coli* that enabled the bacterium to resist the drug [4]. However, given the high association of aminoglycoside resistance to quinolone resistance, it suggests a shared rather than intrinsic mechanism such as plasmid-mediated resistance but the lower overall prevalence of resistance to aminoglycosides shows a smaller contribution of this mechanism in the resistance observed [60, 61].

In this study, we detected plasmid-borne resistance genes to all the three drug classes of choice used against *Enterobacteriaceae*–beta lactams, quinolones and aminoglycosides. There was a high co-occurrence of several resistance genes in the isolates probably indicating the gravity of dissemination of these genes. The case where some isolates tested negative for all the genes tested [in 8/84 (9.5%) ESBL producing isolates, 13/164 (7.9%) quinolones resistant isolates and 7/66 (10.6%) aminoglycosides resistant isolates] can be explained in two ways: first, it may be that the plasmid-borne resistance gene responsible for the resistance phenotype was not tested since the genes tested were not exhaustive; second, it may be that other genetic mechanisms than plasmid mediation such as chromosomal encoded resistance and chromosomal mutations may be responsible [3, 4].

We observed a high prevalence of the beta lactamase genes *blaTEM1* 59/84 (70.24%), *blaCTX-M* 19/84 (22.62%) and *blaKPC* 30/84 (35.71%) of the TEM, CTX and KPC enzyme families in isolates. These are genes for Class A beta lactamase enzymes which are highly disseminated among important *Enterobacteriaceae* pathogens. This is an indication of the risk of their further dissemination and the resistance they confer owing to their occurrence in MGEs and also their ability to expand their spectrum of activity as new antibiotics are developed especially by shuffling of chromosomal genes and mutations [62].

In this study we equally noted a high occurrence of the quinolone resistance genes *qnrA* 65/164 (39.63%) and *qnrB* 34/164 (20.73%). These are clinically important genes to monitor in epidemiological studies because of their enrichment in human-associated environments, mobility, and presence in pathogens [63]. The high occurrence of the *aac(6′)-IB-CR* gene 97/164 (59.15%), though a low level mediator of resistance to ciprofloxacin [64] and the *qnrS* gene 86/164 (51.83%) tie with the high resistance to this antibiotic 142/394 (37.1%) recorded in this study. Worth noting is also the prevalence of the efflux pump mediator gene *qepA* which though being relatively lower, 32/164 (19.51%) is high compared to those recorded in other geographical locations with 0% recorded by Crémet et al. in a French hospital [65] and also by Dahmen et al. in Tunisia [66]; and 0.3% recorded by Yamane et al. in Japan [67]. Though Zhang et al. classified the *qnrS* and *qepA* as low risk gene due to their low dissemination in clinical and human-related environment [63], we note in this study that these genes show a high enrichment in the community studied and correlates with the high resistance to quinolones observed in this study. Thus with this relatively high prevalence they also need to be carefully monitored. PMQR genes showed a high co-occurrence with 132/164 (80.49%) of the isolates positive for more than one PMQR gene, a further indication of their high dissemination. Of the three enzyme classes: N-Acetyltransferases (AAC), O-Adenyltransferases (ANT) and O-Phosphotransferases (APH) involved with PMAR, the *aac(6′)-IB* gene coding for an N-acetyltransferase is the most clinically important gene to monitor in epidemiological studies because of its enrichment in human-associated environments, mobility, and presence in pathogens [63, 68]. This gene had the highest occurrence 51/66 (77.27%) in the isolates tested and this correlates with the high occurrence of its variant, the *aac(6′)-IB-CR* gene 97/164 (59.15%), responsible for PMQR. However, the *aph(3′)-IA* and the ant*(2′)-IA* genes considered non-epidemiologically important because of their previous low enrichment in human-related environment and pathogens [63] showed a much higher prevalence of 23/66 (34.85%) for *aph(3′)-IA* and 14/66 (21.21%) for *ant(2′)-IA*. This indicates that these genes are gradually being enriched in the community and there is need to curb their further dissemination.

These ESBL, PMQR and PMAR genes have also been isolated from human pathogens showing a generalised circulation between humans and animals [33, 53, 56, 69]. This lays emphasis on the need for the extension of the fight against ABR to animal husbandry in Cameroon.

Molecular methods such as the DNA microarray have been developed to simultaneously detect microbes in pathologic samples [70]. It would no doubt have been a powerful tool in our research to detect bacteria with a lot of precision. However, the application of such tools may be limited by cost. Furthermore, determining the different strains of important bacteria with a much higher resolution technique such as the use of Enterobacterial Repetitive Intergenic Consensus Polymerase Chain Reaction (ERIC-PCR) genotyping [71] would have thrown more light into the strains contributing to much of the observed resistance. This will form part of future studies alongside the hypotheses developed through the present investigations.

## 5. Conclusion

This study showed a high carriage of *Enterobacteriaceae* showing phenotypic resistance with corresponding plasmid-borne resistance genes, a widespread and a fairly uniform distribution among broilers in poultry farms across the study area. *Enterobacteriaceae* from chicken in this Region showed high resistance to Penicillins and Tetracyclines and low resistance rates to 3rd generation Cephalosporins and Aminoglycosides. Poor hygienic conditions at poultry farms and rearing chicken for long periods were associated with increased carriage of multi drug resistant *Enterobacteriaceae*. Plasmid-mediated genes for ESBL production, quinolone resistance and aminoglycoside resistance were extensively distributed in both pathogenic and commensal *Enterobacteriaceae* with high co-occurrence of the genes in the isolates. The high prevalence of MDR especially in clinically important genera like *Salmonella*, *Escherichia* and *Klebsiella* indicates the necessity for continuous monitoring and for stakeholders to put efforts and resources to improve sanitation at poultry farms and combat resistance development and misuse of antibiotics in animal farms.

## Figures and Tables

**Table 1:** Antibiotics used.

Class	Antibiotics
Beta lactams	Amoxicillin, amoxicillin/clavulanic acid, ceftriaxone, cefotaxime, ceftazidime and imipenem
Aminoglycosides	Gentamicin and amikacin
Quinolones	Ciprofloxacin and levofloxacin
Tetracycline	Doxycycline

**Table 2:** Oligonucleotides used.

Gene	Primer	Sequence (5'—3')	Size of amplicon		Annealing temperature (°C)	References
ESBL genes						
blaTEM	BLATEM-F	ATAAAATTCTTGAAGACGAAA	1080		53	Farkas, et al. [36].
	BLATEM-R	GACAGTTACCAATGCTTAATC				
blaTEM-1	BLATEM-1-F	GGTCGCCGCATACACTATTC	500		57	
	BLATEM-1-R	ATACGGGAGGGCTTACCATC				
blaTEM-2	BLATEM-2-F	AAGTAAAAGATGCTGAAGATAAGTTGG	737		61	
	BLATEM-2-R	GATCTGTCTATTTCGTTCATCCATAG				
blaCTX-M	BLACTX-M-F	GTGAAACGCAAAAGCAGCTG	400		61	
	BLACTX-M-R	CCGGTCGTATTGCCTTTGAG				
blaSHV-1	BLASHV-1-F	GCGTTATATTCGCCTGTGTATTAT	385		58	
	BLASHV-1-R	GCCTGTTATCGCTCATGGTAATG				
blaKPC	BlaKPC F	TGTCACTGTATCGCCGTC	900		58	Mosca, et al. [37]
	BlaKPC R	CTCAGTGCTCTACAGAAAACC				

PMQR genes						
qnrA	QNRA-F	TCAGCAAGAGGATTTCTCA	627		58	Minh Vien et al. [38].
	QNRA-R	GGCAGCACTATTACTCCCA				
qnrB	QNRB-F	GATCGTGAAAGCCAGAAAGG	476		58	
	QNRB-R	ACGATGCCTGGTAGTTGTCC				
qnrS	QNRS-F	ATGGAAACCTACAATCATAC	491		58	
	QNRS-R	AAAAAACACCTCGACTTAAGT				
aac(6')IB-CR	AAC(6')IB-CR-F	TTGCGATGCTCTATGAGTGGCTA	482		58	
	AAC(6')IB-CR-R	CTCGAATGCCTGGCGTGTTT				
qepA	QEPA-F	GCAGGTCCAGCAGCGGGTAG	199		60	
	QEPA-R	CTTCCTGCCCGAGTATCGTG				

PMAR genes						
aac(6')-IB	aac(6')-Ib—F	AGTACTTGCCAAGCGTTTTAGCGC		365	58	Kim, et al. [39].
	aac(6')-Ib—R	CATGTACACGGCTGGACCAT				
aph(3')-IA	aph(3')-Ia—–F	ATGGGCTCGCGATAATGTCG		734	57	
	aph(3')-Ia—R	AGAAAAACTCATCGAGCATC				
ant(2')-IA	ant(2')-Ia—F	ATGCAAGTAGCGTATGCGCT		477	57	
	ant(2')-Ia—R	TCCCCGATCTCCGCTAAGAA				

**Table 3:** Colony aspect and biochemical reactions of the isolated organisms.

	Colony on EMB agar	Glucose	Lactose	H_2_S	Gas	Urease	Indole	Mannitol	Motility	Catalase	Citrate
*Escherichia*	Small, flat, dark violet with or without a metallic green sheen in reflected light	+	+	−	+	−	+	+	+	+/−	−
*Salmonella*	Translucent, amber coloured	+	−	+	+/−	−	−	+	+	+	+−
*Klebsiella*	Large, pink and mucoid	+	+	−	+	+	+/−	+	−	+	+
*Proteus*	Translucent	+	−	+/−	+/−	+	+/−	−	+	+	+/-
*Citrobacter*	Small, flat, dark violet with or without a metallic green sheen in reflected light	+	+	+/-	+	−	−	+	+	+	+
*Enterobacter*	Pink, dark centre	+	+	−	+	−	−	+	+	+	+
*Providencia*	Colourless smooth and translucent	+	−	−	+/-	−	+	+	+	+	+
*Hafnia*	Large, transparent and circular	+	−	−	+	−	−	+	+	+	−
*Shigella*	Translucent, amber coloured	+	−	−	−	−	+/−	+	−	+	−
*Raoultella*	Light pink	+	+	−	−	+	+	+	−	+	+
*Yersinia*	Transparent, colourless with no zone of precipitation	+	−	−	−	+	+/−	+	+	+	−
*Morgenella*	Flat, translucent	+	−	−	+	+	+	+	+	+	−

**Table 4:** Locations of farms, number of samples and isolates and the risk factors to which chicken subjects were exposed.

Location	Number of farms	Samples (1 per subject)	Isolates	Evaluated risks at farms				
				Use of antibiotics	Age of subject	Poor feeding hygiene	Unsure water	Poor sanitation
Bafoussam Rural	4	40	66	4	18, 22	3/4	¼	2/4
Bafoussam Urban	5	50	72	5	22, 27	2/5	0	2/4
Batcham	1	10	16	0	5, 5	1	1	1
Balesseng	2	15	22	2	8, 7	2	2	1/2
Banjoun	3	30	44	3	18, 12	2/3	2/3	1/3
Dschang	3	30	40	3	16, 14	2/3	2/3	3
Foumbot	2	20	26	1/2	11, 9	2	½	2
Mbouda	2	20	24	2	11, 9	2	1/2	1/2
Nkong-Ni	2	20	27	2	10, 10	2	2	2
Penka Michel	2	20	27	2	10, 10	1/2	2	1/2
Santchou	2	20	30	2	10, 10	1/2	2	2
Total	28	275	394	26/28	138, 137	20/28	16/28	18/28
Percentage exposure to risk				92.86	50.18, 49.81	71.43	57.14	64.29

Evaluated risk	Description							
Unsure water	Using any other source of water apart from pipe-borne water without prior treatment.							
Use of antibiotics	Use of antibiotics in feed for chicken subjects.							
Sanitation	Conditions of the environment such as litter, stagnant sewage, rearing of animals around poultry farm, state of workers' restroom.							
Feeding hygiene	Cleaning of feeders and drinkers at least twice a week.							
Age	≤30 days for category “young” and ˃30 days for category “old” chicken.							

Bacterial carriage per sample.

**Table 5:** Relationship between bacterial carriage in samples and environmental risk factors.

	Bacteria carriage (number of colonies) of ≥3 per sample	
	Odds ratio (95% CI)	*p* value (*significant correlation ≤0.05*)
Poor feeding hygiene	2.55 (1.67, 3.89)	0.001
Unsure water	1.75 (1.16, 2.64)	0.011
Poor sanitation	1.97 (1.31, 2.96)	0.009

**Table 6:** Prevalence in percentage of isolates by location.

Location organism	Penka Michel	Batcham	Bafoussam Rural	Bafoussam Urban	Bandjoun	Dschang	Santchou	Nkong-Ni	Foumbot	Mbouda	Balesseng	Mean	Standard deviation	Coefficient of Variation
*Citrobacter*spp	6.70	4.76	00	22.73	7.84	17.24	6.25	4.55	3.85	8.82	0.00	7.5	6.6	0.9
*Enterobacter*spp	3.45	00	33.33	4.54	5.88	17.24	9.38	9.09	11.54	5.82	12.50	10.3	8.6	0.8
*Escherichia*spp	31.03	33.33	16.67	18.18	9.80	17.24	21.88	31.81	19.23	20.59	25.00	22.3	7.0	0.3
*Hafnia*spp	13.79	00	16.67	13.64	3.92	3.45	3.13	4.55	0.00	2.94	4.17	6.0	5.5	0.9
*Klebsiella*spp	00	4.76	16.67	4.54	3.92	3.45	12.50	18.18	19.23	11.76	12.50	9.8	6.4	0.7
*Morgenella*spp	3.45	00	00	00	00	00	6.25	0.00	3.85	5.82	4.17	2.1	2.5	1.2
*Proteus*spp	6.72	9.52	2.35	3.68	15.69	3.45	6.25	9.09	15.38	14.71	12.50	9.0	4.7	0.5
*Providencia*spp	13.79	9.52	00	4.54	15.69	6.70	3.13	4.55	3.85	2.94	4.17	6.3	4.6	0.7
*Raoultella*spp	00	4.76	00	4.54	3.92	3.45	6.25	0.00	3.85	5.82	4.17	3.3	2.2	0.7
*Salmonella*spp	13.79	19.05	16.67	18.18	29.41	13.79	15.63	18.18	15.38	14.71	16.67	17.4	4.2	0.2
*Shigella*spp	3.45	9.52	00	0	3.92	3.45	6.25	0.00	3.85	2.94	4.17	3.4	2.7	0.8
*Yersinia*spp	3.45	4.76	00	9.09	00	10.34	3.13	0.00	0.00	2.94	0.00	3.1	3.6	1.2

**Table 7:** Prevalence in percentage of antibiotic resistance in various isolates.

Organisms	Resistant to beta lactams					Resistant to aminoglycosides		Resistant to quinolones		Resistant to tetracycline	Showing MDR	Showing ESBL
	Penicillins		Cephalosporins		Carbapenem	Gentamicin	Amikacin	Ciprofloxacin	Levofloxacin	Doxycycline		
	Amoxicillin	Amoxicillin/clavulanic acid	Ceftriaxone	Cefotaxime	Imipenem							
*Citrobacter*spp	88.24	44.12	8.82	8.82	8.82	8.82	2.94	58.82	50.00	97.06	47.06	14.71
*Enterobacter*spp	87.10	58.06	9.68	9.68	0.00	6.45	3.23	22.58	22.58	96.77	45.16	19.35
*Escherichia*spp	93.83	58.02	18.52	14.81	3.70	16.05	4.94	64.20	56.79	92.59	55.56	24.69
*Hafnia*spp	84.21	63.16	31.58	10.53	0.00	31.58	5.26	26.32	36.84	89.47	47.37	21.05
*Klebsiella*spp	92.31	61.54	17.95	17.95	5.13	15.38	2.56	23.08	23.08	94.87	38.46	30.77
*Morgenella*spp	100.00	62.50	0.00	12.50	0.00	12.50	0.00	25.00	25.00	100.00	12.50	25.00
*Proteus*spp	89.47	55.26	18.42	10.53	5.26	13.16	7.89	50.00	44.74	84.21	47.37	21.05
*Providencia*spp	82.14	53.57	7.14	14.29	3.57	21.43	7.14	32.14	17.86	92.86	46.43	25.00
*Raoultella*spp	100.00	71.43	21.43	14.29	0.00	35.71	14.29	21.43	14.29	100.00	42.86	14.29
*Salmonella*spp	82.43	54.05	14.86	9.46	8.11	13.51	2.70	27.03	25.68	94.59	47.30	16.22
*Shigella*spp	100.00	73.33	20.00	20.00	0.00	26.67	0.00	26.67	26.67	93.33	46.67	20.00
*Yersinia*spp	115.38	76.92	15.38	15.38	0.00	15.38	0.00	30.77	23.08	100.00	38.46	23.08
Overall	**345 (87.8%)**	**227 (57.8%)**	**79 (20.1%)**	**65 (16.5%)**	**16 (4.1%)**	**58 (14.5%)**	**12 (3.1%)**	**142 (37.1%)**	**124 (33.1%)**	**380 (96.7%)**	**173 (44.0%)**	**84 (21.32%)**

**Table 8:** Association of risks to resistance outcomes.

Factor	Outcome	Risk estimate			Correlation(*significant correlation ≤* *0.05*)
		Odds ratios (*increased risk > 1*)	95% confidence interval		
			Lower	Upper	
Use of antibiotics	Resistance	1.394	0.852	2.280	0.186
Age of chicken	Resistance	13.491	8.274	21.997	0.001
Feeding hygiene	Resistance	1.783	1.172	2.714	0.007
Sanitation	Resistance	1.495	1.000	2.234	0.049
Unsure water	Resistance	0.709	0.471	1.065	0.098
Use of antibiotics	ESBL	0.669	0.356	1.259	0.212
Age of chicken	ESBL	4.505	2.352	8.626	0.001
Feeding hygiene	ESBL	1.182	0.657	2.125	0.578
Sanitation	ESBL	1.322	0.754	2.316	0.330
Unsure water	ESBL	2.589	1.372	4.885	0.003
Use of antibiotics	MDR	1.490	0.897	2.473	0.123
Age of chicken	MDR	7.980	5.054	12.600	0.001
Feeding hygiene	MDR	1.538	1.004	2.354	0.047
Sanitation	MDR	1.348	0.900	2.018	0.148
Unsure water	MDR	0.544	0.362	0.818	0.003

**Table 9:** Prevalence of plasmid-borne beta lactamase genes detected by PCR.

Plasmid-borne ESBL genes						
—	*blaTEM*	*blaTEM-1*	*blaTEM-2*	*blaCTX-M*	*blaSHV-1*	*blaKPC*
Total showing ESBL production	84					
Positive for gene (%)	27 (32.14)	59 (70.24)	16 (19.05)	19 (22.62)	10 (11.90)	30 (35.71)
*E. coli* showing ESBL production	15					
*E. coli* positive for gene (%)	5 (33.33)	14 (93.33)	5 (33.33)	4 (26.67)	2 (13.33)	7 (46.67)
*Klebsiella* showing ESBL production	7					
Klebsiella positive for gene (%)	2 (28.57)	5 (71.43)	1 (14.29)	2 (28.57)	1 (14.29)	4 (56.14)
*Salmonella* showing ESBL production	10					
*Salmonella* positive for gene (%)	4 (40.00)	8 (80.00)	3 (30.00)	3 (30.00)	1 (10.00)	4 (40.00)
Others showing ESBL production	52					
Others positive for gene (%)	16 (30.77)	32 (61.54)	7 (13.46)	10 (19.23)	6 (11.54)	14 (26.92)

**Table 10:** Prevalence of PMQR genes detected by PCR.

PMQR genes					
—	*qnrA*	*qnrS*	*qnrB*	*aac(6')-IB-CR*	*qepA*
Total showing resistance to quinolones	164				
Positive for gene (%)	65 (39.63)	86 (51.83)	34 (20.73)	97 (59.15)	32 (19.51)
*E. coli* showing resistance to quinolones	52				
*E. coli* positive for gene (%)	24 (46.15)	27 (51.92)	9 (17.30)	30 (57.70)	10 (19.23)
*Klebsiella* showing resistance to quinolones	9				
Klebsiella positive for gene (%)	4 (44.44)	5 (55.55)	3 (33.33)	5 (55.55)	3 (33.33)
*Salmonella* showing resistance to quinolones	20				
*Salmonella* positive for gene (%)	4 (20.00)	11 (55.00)	4 (20.00)	10 (50.00)	3 (15.00)
Others showing resistance to quinolones	83				
Others positive for gene (%)	34 (40.96)	44 (53.01)	18 (21.69)	52 (62.65)	17 (20.48)

**Table 11:** Prevalence of PMAR genes detected by PCR.

PMAR genes			
—	*aph(3´)-IA*	*ant(2´)-IA*	*aac(6´)-IB*
Total showing resistance to aminoglycosides	66		
Positive for gene (%)	23 (34.85)	14 (21.21)	51 (77.27)
*E*. *coli* showing resistance to aminoglycosides	11		
*E. coli* positive for gene (%)	6 (54.55)	2 (18.18)	9 (81.82)
*Klebsiella* showing resistance to aminoglycosides	10		
Klebsiella positive for gene (%)	3 (30.00)	1 (10.00)	5 (50.00)
*Salmonella* showing resistance to aminoglycosides	9		
*Salmonella* positive for gene (%)	4 (44.44)	3 (33.33)	8 (88.89)
Others showing resistance to aminoglycosides	36		
Others positive for gene (%)	10 (27.78)	8 (22.22)	28 (77.78)

**Table 12:** Co-occurrence of resistance genes in isolates.

	Total number of isolates tested	Number of isolates not positive for any of the resistance genes tested	Number of isolates positive to only 1 resistance gene tested	Number of isolates positive to 2 resistance genes tested	Number of isolates positive to 3 resistance genes tested	Number of isolates positive to 4 resistance genes tested	Number of isolates positive to 5 resistance genes tested
ESBL resistance genes	84	8 (9.5%)	18 (21.4%)	33 (39.3%)	21 (25.0%) 21	4 (04.8%)	—
Quinolone resistance genes	164	13 (7.9%)	19 (11.6%)	83 (50.6%)	38 (23.2%)	11 (06.7%)	—
Aminoglycoside resistance genes	66	6 (10.6%)	32 (48.5%)	24 (36.4%)	4 (06.1%)	—	—

Taking total isolates minus isolates with one or no gene detected, this table shows that there was co-occurrence of plasmid-borne genes in 58/84 (69.05%) of ESBL producers, 132/164 (80.49%) of quinolone resistant isolates and 28/66 (42.42%) of aminoglycoside resistant isolates.

## Data Availability

The numerical data used to support the findings of this study are included in the article.
